# Presumptive malignant transformation of chronic polypoid cystitis into an apical transitional cell carcinoma without BRAF mutation in a young female dog

**DOI:** 10.1111/jvim.16107

**Published:** 2021-03-19

**Authors:** Emmanuelle Marie Butty, Shelley Hahn, Mary Anna Labato

**Affiliations:** ^1^ Tufts University Cummings School of Veterinary Medicine Internal Medicine North Grafton Massachusetts USA

**Keywords:** bladder, carcinoma in situ, urology, urothelial carcinoma

## Abstract

A 3‐year‐old spayed female English Springer Spaniel was presented twice 4 months apart for investigation of hematuria and pollakiuria without urinary tract infection. Both ultrasound examinations identified a stable craniodorsal bladder wall thickening. The first cystoscopic biopsy samples indicated lymphoplasmacytic cystitis and the second polypoid cystitis. The dog was represented 8 months later for recurrent clinical signs despite medical management. Although the ultrasound examination showed stable disease, repeat cystoscopic biopsy identified transitional cell carcinoma (TCC), confirmed on tissue removed by partial cystectomy. No BRAF mutation was ever detected in urine or tissue samples. To our knowledge, this case represents the first report of presumptive malignant transformation of polypoid cystitis into an apical TCC in a dog. Dogs with polypoid cystitis should be followed closely and surgical management considered if rapid resolution is not achieved with medical management.

**Abbreviations**
CIS: carcinoma in situ
hpf: high‐power field
RBC: red blood cells
TCC: transitional cell carcinoma
UC: urothelial carcinoma
UTI: urinary tract infection
WBC: white blood cells

## 1 INTRODUCTION

Polypoid cystitis is a common disease of the urinary bladder in dogs.^1^, ^2^ The cause of polyp formation in dogs remains unknown. It may represent an inflammatory and hyperplastic reaction to chronic irritation of the bladder mucosa,^3^ and has been most commonly associated with chronic urinary tract infection (UTI) and urolithiasis.^3^, ^4^, ^5^, ^6^, ^7^, ^8^, ^9^, ^10^, ^11^ In human medicine, polypoid cystitis is believed to be an inflammatory reaction to injury most commonly caused by longstanding indwelling catheterization.^12^, ^13^, ^14^, ^15^ Despite its common occurrence, limited information on polypoid cystitis in dogs has been published.^3^, ^4^, ^5^, ^6^, ^7^, ^8^, ^9^, ^10^, ^11^ In 1 case, polypoid cystitis developed after cystoscopic‐guided laser ablation of ectopic ureters.^16^

Macroscopically, the mucosa is thrown into broad‐based folds, often projecting into the bladder lumen, covering a core of proliferating stroma. Occasionally, no macroscopic lesions or a diffuse thickening of the bladder wall is observed rather than a defined mass.^3^ Microscopically, epithelial cords project down into the underlying stroma, accompanied by mucosal erosions and ulcerations, stromal edema, inflammation, and hemorrhage.^1^, ^2^, ^3^, ^17^ Dysplastic changes of the epithelium, characterized by nuclear atypia and increased mitotic activity, are found in some cases.^2^, ^3^

Polypoid cystitis is an important differential diagnosis of bladder cancer both in people and in dogs, and histopathology confirms a final diagnosis in most cases. Liquid biopsy using the CADET BRAF test is a new noninvasive diagnostic tool to detect cells and DNA released by transitional cell carcinoma (TCC) in dogs with a sensitivity and specificity of more than 95% and 100%, respectively.^18^, ^19^, ^20^, ^21^

In human medicine, polypoid cystitis and other types of hyperplastic cystitis often are accompanied by metaplasia and dysplasia of the epithelium and are considered preneoplastic changes.^17^, ^22^, ^23^. In veterinary medicine, however, the clinical relevance of such changes as preneoplastic is not well established, and a study found such changes could be normal findings in the canine urinary bladder.^24^ Despite rare reports of neoplastic transformation of polypoid cystitis, the biologic behavior of polypoid cystitis and its neoplastic potential have not yet been elucidated.^3^, ^5^, ^10^

## 2 CASE DESCRIPTION

A 3‐year‐old spayed female English Springer Spaniel was referred to the Foster Hospital for Small Animals at the Cummings Veterinary Medical Center at Tufts University for investigation of hematuria and pollakiuria. The dog had been presented 2 weeks earlier to the primary care veterinarian with a 4‐day history of excessive licking of the vulva. Ultrasound examination showed an irregular bladder wall. The urine was dark yellow and urinalysis showed a specific gravity of 1.035, mild proteinuria (1+), and hematuria (>50 red blood cells [RBC]/high‐power field [hpf]) without pyuria (2‐3 white blood cells [WBC]/hpf) and no bacteria. A CBC and serum biochemistry profile were normal, and urine culture was negative. The dog was treated with enrofloxacin (7.3 mg/kg PO q12h for 7 days), which resolved the excessive licking and normalized the color of the urine. The dog was presented 1 month later to the primary care veterinarian for evaluation of pollakiuria. Focal ultrasound examination showed persistent irregular bladder wall thickening and the dog was referred for further investigation.

**FIGURE 1:**
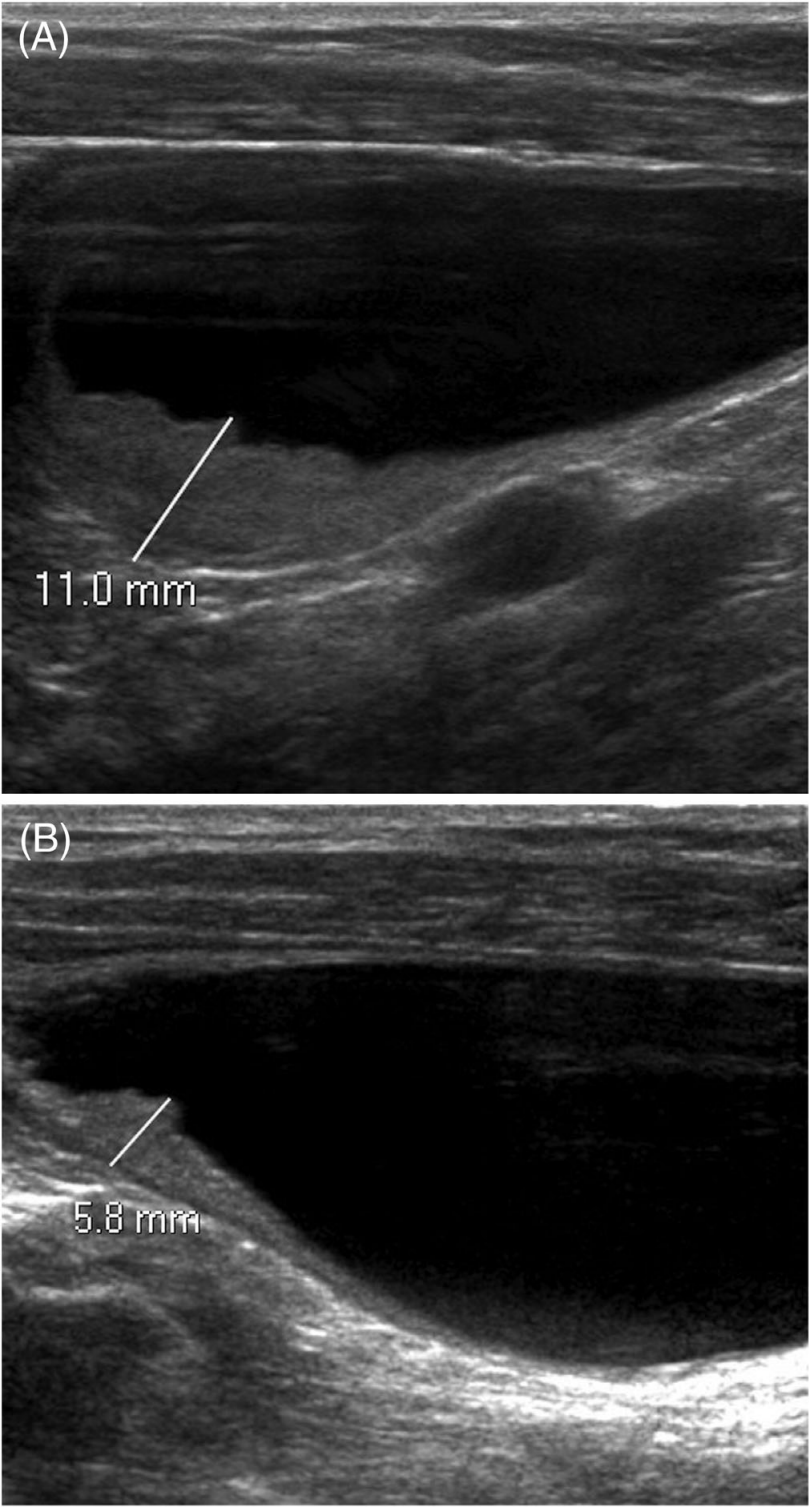
Ultrasonographic examinations before (A) and 10 weeks after (B) initial treatment with a 6‐week course of marbofloxacin and meloxicam

**FIGURE 2:**
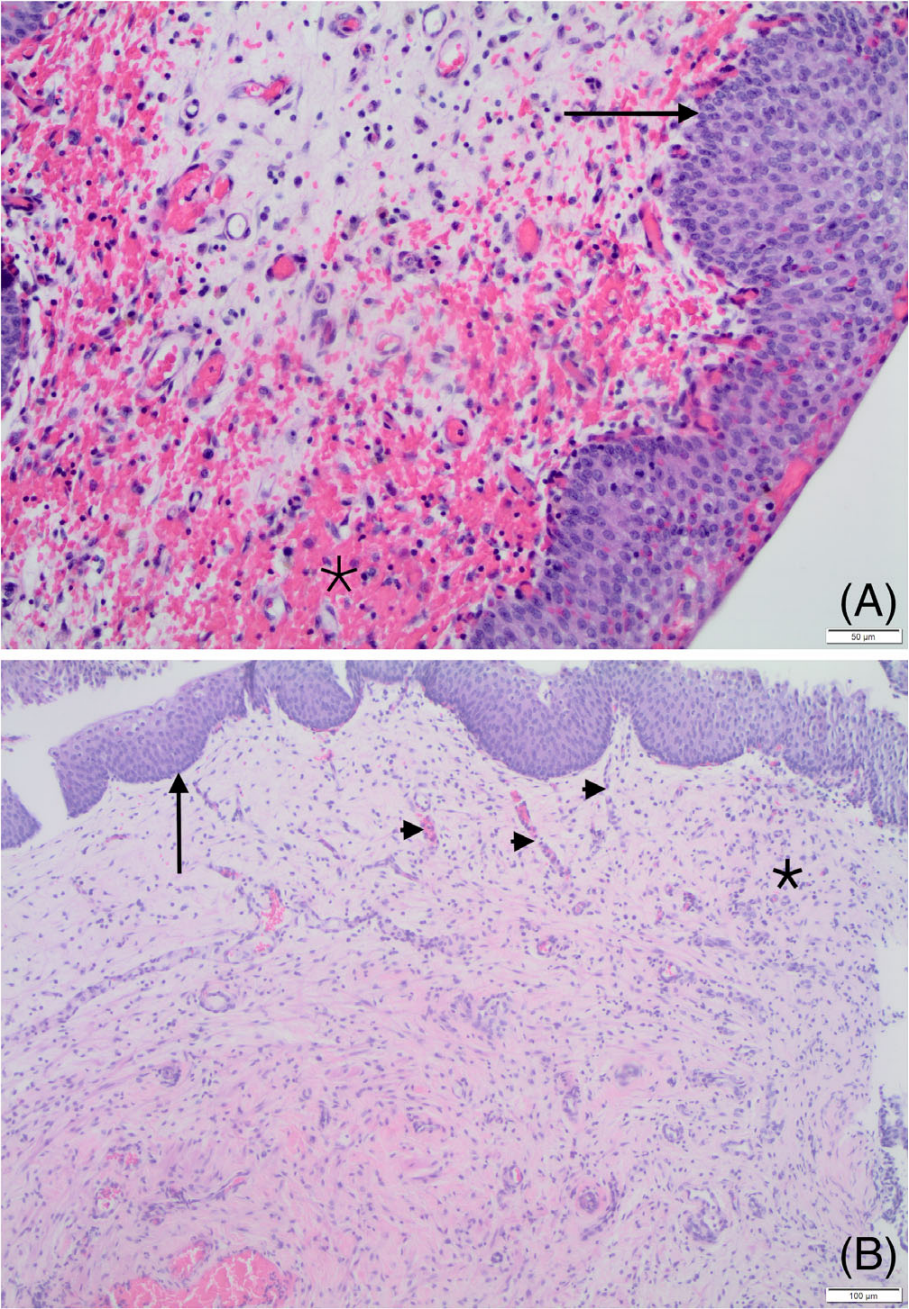
Histopathology of the first biopsy. A, Proprial hemorrhage and inflammation (asterisk) and mucosal hyperplasia (arrow). B, Proprial inflammation (asterisk) and mucosal hyperplasia (arrow). Note the vertically oriented blood vessels indicating granulation tissue (arrowhead)

**FIGURE 3:**
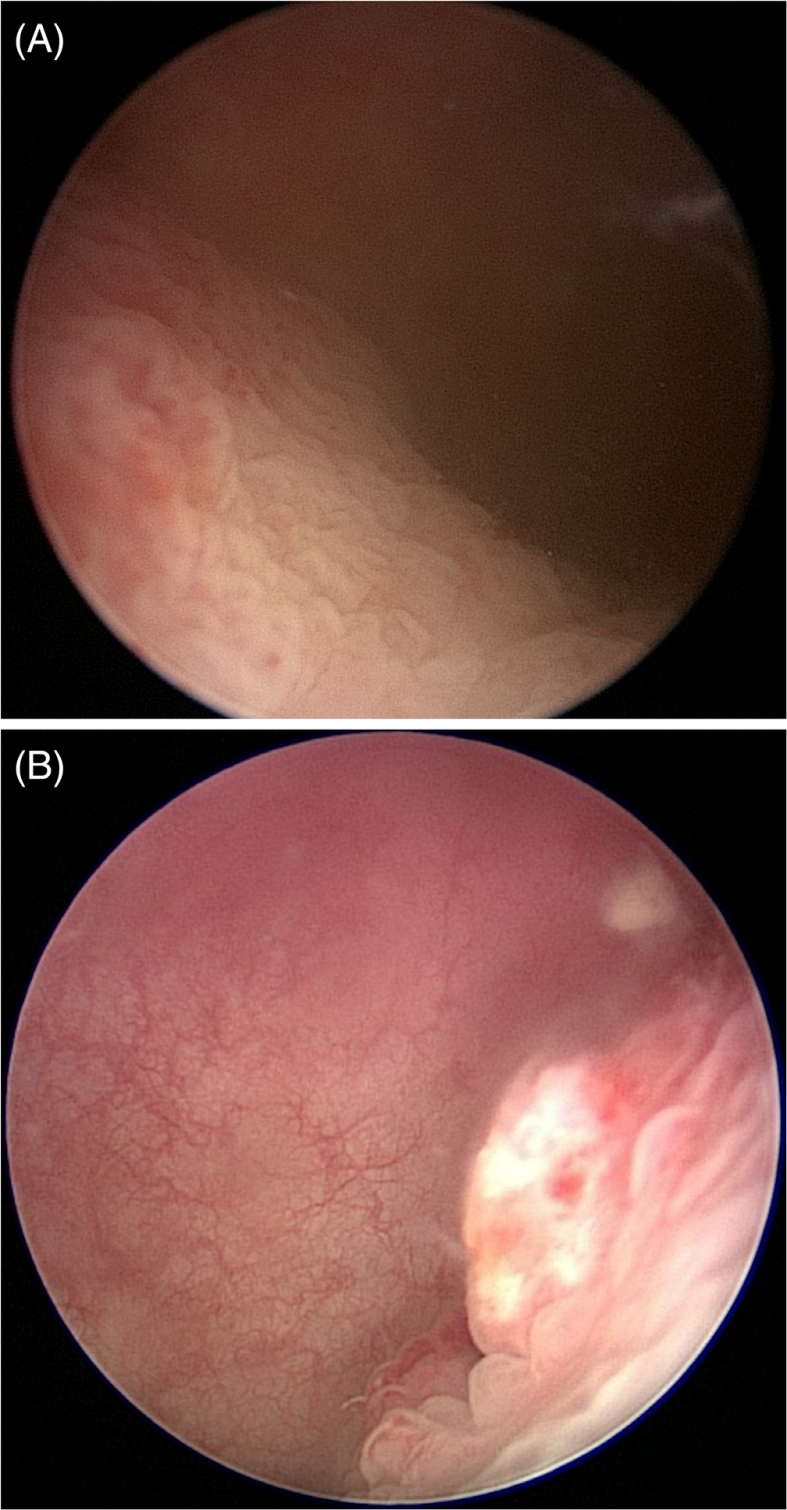
Evolution of the bladder changes on cystoscopic examinations. Interventions between cystoscopies included administration of meloxicam and marbofloxacin. A, Initial examination: friable thickened and irregular dorsal bladder wall with mild erosions. B, Images 3.5 months after the initial examination: dorsal bladder wall thickening with irregular mucosa, mild erosions, and mineralization

At the time of presentation to the referral hospital 1 week later, the patient had developed hematuria in addition to pollakiuria. Abdominal ultrasound examination identified an extensive craniodorsal bladder wall thickening (up to 11 mm; Figure 1). Thoracic radiographs were normal. Urinalysis showed a specific gravity of 1.018 and pH of 8 with trace of proteinuria, hematuria (10‐20 RBC/hpf) and pyuria (20‐30 WBC/hpf). Urine culture was negative. Cystoscopy examination identified thickening of the dorsal bladder wall and friable mucosa with multifocal, pinpoint erosions. The patient was discharged on a 7‐day course of amoxicillin/clavulanate (13.5 mg/kg PO q12h) and meloxicam (0.08 mg/kg PO q24h). Seven cystoscopic biopsy specimens were collected. The samples included mucosa and lamina propria from a single polyp and showed diffuse, marked lymphoplasmacytic and neutrophilic cystitis with lamina propria hemorrhage and mucosal hyperplasia (Figure 2). No BRAF mutation was detected using the CADET BRAF test (detection threshold, 0.39%). Two weeks later, the dog was presented again for persistent pollakiuria. On urinalysis, specific gravity was 1.024 with mild proteinuria (1+), pyuria (>50 WBC/hpf), and hematuria (21‐50 RBC/hpf) and >100 rod‐shaped bacteria/hpf. A CBC and serum biochemistry profile were normal. Repeat cystoscopy identified a persistently friable, thickened, and irregular dorsal bladder wall with mild erosions (Figure 3). Both urine and bladder wall were cultured and an *Escherichia coli* sensitive to all antibiotics tested was isolated. The dog was prescribed a 6‐week course of marbofloxacin (4 mg/kg PO q24h) and meloxicam (0.08 mg/kg PO q24h) with normal urinalysis and negative urine culture findings while on both medications. Treatment completely resolved hematuria and pollakiuria, and only rare episodes of nocturia were reported.

Ten weeks later, the dog was presented for repeat ultrasound examination. The urinary bladder wall was markedly improved but remained slightly thickened cranially (5.8 mm) with slightly irregular mucosa (Figure 1). Urine specific gravity was 1.014 and the reminder of the urinalysis was normal. Urine culture was negative.

**FIGURE 4:**
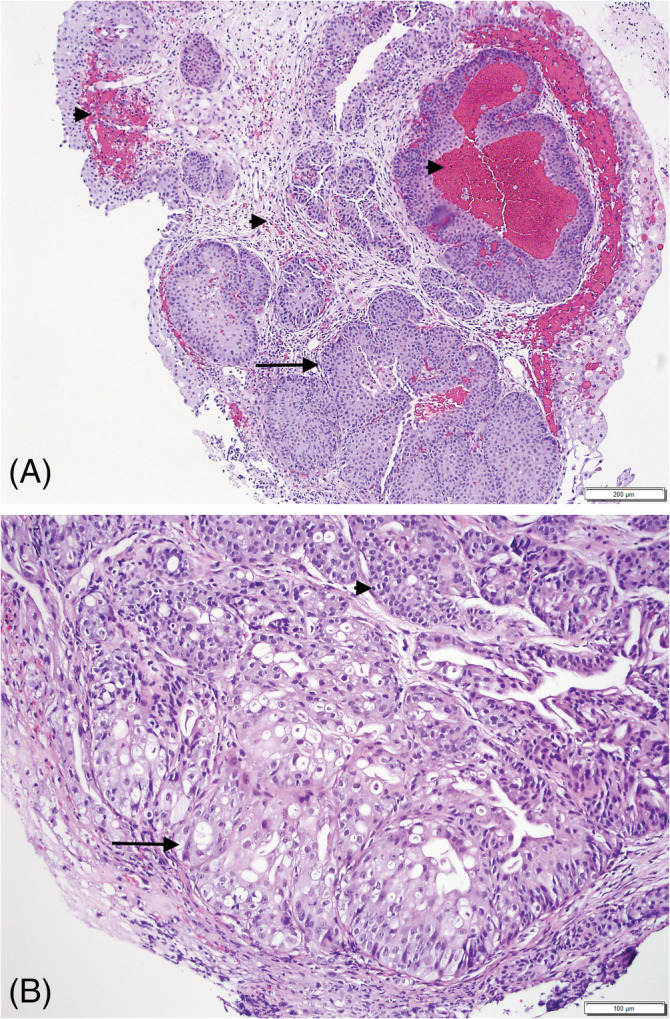
Histopathology of the second biopsy. A, Polypoid mucosal hyperplasia (arrow) and proprial inflammation and hemorrhages (arrowhead). B, Another area from the same biopsy showing low grade urothelial carcinoma: Islands of uroepithelium, showing hypereosinophilia, cystic degeneration and vesicular nuclei (arrow) alongside smaller cells, with minimal cellular and nuclear atypia (arrowhead)

One month later, the dog became markedly pollakiuric, and was treated with marbofloxacin (4 mg/kg PO q24h) by the primary veterinarian pending a urine culture, which was negative. A repeat ultrasound examination showed that the urinary bladder wall was still slightly thickened cranially (5.8 mm) with slightly irregular mucosal margins. Cystoscopy identified dorsal bladder wall thickening with irregular mucosa, mild erosions, and mineralization (Figure 3). Bladder wall culture was negative. Microscopic examination of biopsy samples showed thickened urothelial mucosa (up to 2‐3 times normal thickness) with broad‐based projections expanding the underlying propria, occasionally associated with multifocal erosions or ulcerations. A mixture of lymphocytes, plasma cells, and a few eosinophils expanded the lamina propria, accompanied by edema and hemorrhage (Figure 4). Most of the epithelial cells exhibited no cellular or nuclear atypia and no mitotic activity (Figure 4A). In a few areas, however, the epithelial cells were hypereosinophilic and exhibited cystic degeneration, anisocytosis, and anisokaryosis (Figure 4B). The nuclei assumed a vesicular appearance, nucleoli were prominent and mitotic figures were evident (4/hpf). Periodic acid‐Schiff stain identified PAS‐positive, rare intracytoplasmic, and eosinophilic bodies (Melamed‐Wollinska bodies). The CADET BRAF and CADET BRAF Plus tests were repeated but no BRAF mutation was detected (detection threshold, 0.08%). This biopsy sample originally was diagnosed as low‐grade TCC, but was rediagnosed as polypoid cystitis by the same pathologist who reviewed the first biopsy sample. The dog then was followed by the primary care veterinarian and clinical signs were managed using a daily dose of meloxicam (0.1 mg/kg PO q24h).

The dog was first presented to us 8 months later for worsening pollakiuria. Marbofloxacin (4 mg/kg PO q24h) had been restarted recently by the primary care veterinarian without improvement of the clinical signs. On abdominal ultrasound examination, the previously observed dorsal urinary bladder wall thickening appeared similar, but more polypoid, with 3 discrete foci of mixed echogenicity projecting into the bladder lumen. Thoracic radiographs and repeated CBC and serum biochemistry profile were normal. On cystoscopy, the dorsal bladder wall appeared thickened and irregular with small erosions and 3 polypoid structures. Multiple biopsy samples including the polypoid structures, which were easily removed at their base, were submitted for culture and histopathology. The bladder wall culture was negative. The CADET BRAF and CADET BRAF Plus tests were repeated, but again no BRAF mutation was detected (detection threshold, 0.118%).

**FIGURE 5:**
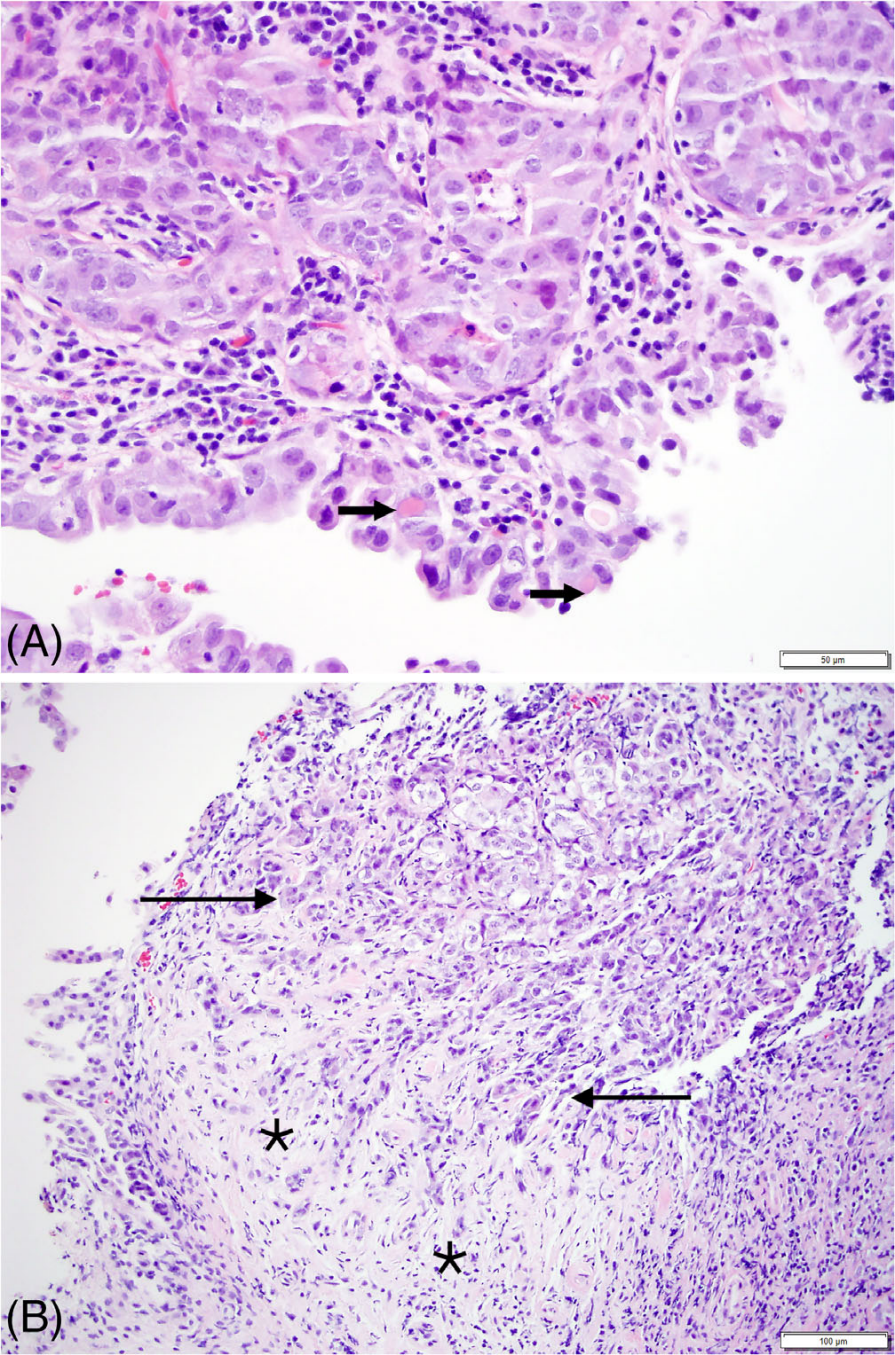
Evolution of the bladder changes on histopathologic examinations 8 months after the second biopsies. Transitional cell carcinoma: A, the neoplastic cells exhibit anisokaryosis and anisocytosis, with intracytoplasmic “Melamed‐Wolinska” bodies (arrows). B, Neoplastic urothelium infiltrating in irregular fronds into the underlying propria (arrow), and individual cells embedded in scirrhous (desmoplastic) fibrous tissue (asterisk)

Microscopically, the samples consisted of fragments of urinary epithelium with superficial lamina propria expanded and replaced by a neoplastic cell population arranged in irregular islands (Figure 5) embedded in a dense stroma (Figure 5B). The neoplastic cells had indistinct borders with abundant eosinophilic cytoplasm and intracytoplasmic vacuoles containing eosinophilic fluid (cystic degeneration). Prominent vesicular nuclei with multiple nucleoli were observed in most of the cells. Anisocytosis and anisokaryosis were marked and mitotic index was 16/10 hpf, with atypical mitoses. Abundant lymphocytic infiltrates accompanied the neoplastic population. In a few sections, the previously diagnosed polypoid cystitis still was evident. The final diagnosis of 2 new pathologists was TCC. This diagnosis was based on the presence of criteria of malignancy in the epithelial cells, and scirrhous stroma, a common phenomenon in carcinomas, in which neoplastic cells induce proliferation of fibroblasts and deposition of collagen.

**FIGURE 6:**
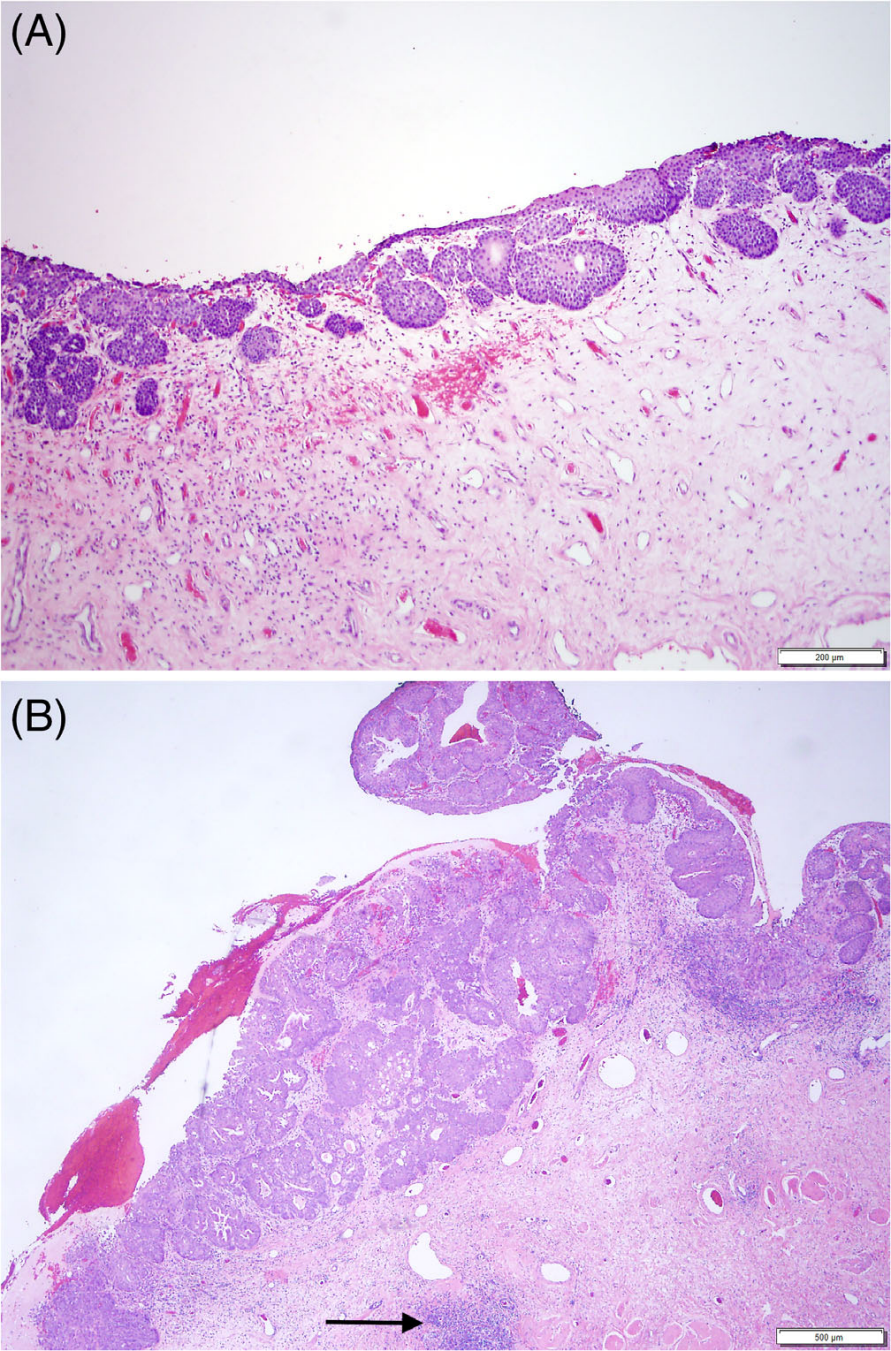
Partial cystectomy histopathology. A, Polypoid cystitis is still visible in this section, accompanied by typical changes: hemorrhage, proprial edema, and inflammation. B, Area of transition from the same biopsy, showing development of TCC (on the left) adjacent to polypoid proliferation of the mucosa (on the right). Note invasion into the lamina propria of neoplastic epithelium. A lymphoid follicle is observed as well, a common finding in polypoid cystitis (arrow)

Because of the location of the bladder lesions, the dog underwent partial cystectomy 1 week later. Microscopic examination performed by the same 2 pathologists confirmed TCC and concurrent polypoid cystitis (Figure 6). The narrowest distance from neoplastic cells to a surgical border was approximately 1 mm. A research case BRAF analysis (Antech Molecular Innovations LLC, Cary, North Carolina) was performed on bladder wall tissue removed by partial cystectomy. No BRAF mutation was detected (detection threshold, 0.06%), and the CADET BRAF Plus did not detect the presence of additional genomic signatures of TCC.

## 3 DISCUSSION

To the best of our knowledge, this represents the first case report of a young dog with presumptive malignant transformation of polypoid cystitis into TCC without BRAF mutation.

In human medicine, some polypoid lesions eventually undergo neoplastic transformation, but the causative factors are not clear.^25^ Inactivation of tumor suppression genes p53 and fragile histidine triad (FHIT) occurs in the majority of bladder TCCs in humans,^2^, ^25^ and some studies have shown that p53 is also suppressed in some types of cystitis.^25^ However, information on genetic mutations in bladder lesions of dogs is lacking. The malignant potential of polypoid cystitis and bladder polyps in veterinary medicine previously has been suspected but never confirmed, and whether mutations in benign polypoid lesions can lead to neoplasia is unknown.^3^, ^5^, ^10^ There are rare reports in the veterinary literature of polypoid cystitis or bladder polyps that have presumably progressed to urothelial carcinoma (UC),^3^, ^5^ but none has done so over the course of 1 year. Additional studies are needed to determine the relationship between chronic cystitis and development of TCC.^3^ Presently, we assume that UC in dogs progresses through stages of carcinoma in situ (CIS), low grade to high grade, and that we only recognize the tumor when it is advanced.^2^

In our case, the young age of the dog and location of the TCC are unusual. The most common location of UC in dogs is the trigone area of the urinary bladder.^2^ Urothelial carcinoma is a neoplasm of older dogs (average age, 9‐11 years)^2^, ^26^, ^27^ and is most common in females.^2^, ^26^ Polypoid cystitis has been reported to arise from the cranioventral bladder mucosa in most cases.^3^ Younger male dogs are reported to be the predominant patient group in some studies,^2^ whereas in the only case series (17 dogs), a strong female predisposition was found, and ages ranged from 2 to 13 years.^3^

On ultrasound examination, at cystoscopy, or on gross examination, polypoid cystitis may be mistaken for a urothelial neoplasm.^2^, ^3^, ^17^ The mucosal surface of the bladder is elevated by a single polyp or multiple nodular to polypoid lesions protruding into the lumen. On cut surface, the proliferations do not extend below the mucosa.^2^ Microscopically, the fronds of polypoid cystitis typically are much broader than those of a papillary carcinoma^17^ and the urothelium is hyperplastic, but usually not stratified as in carcinomas.^17^ In addition, the fibrovascular cores of the papillae in TCC typically lack the prominent inflammation that characterizes both papillary and polypoid cystitis, and the edema seen in the latter, at least in human patients.^17^ Lymphoid aggregates may be prominent in both polypoid lesions and UC.^1^, ^2^, ^17^, ^26^, ^27^

Crucial to the diagnosis of TCC in dogs are location in the urinary bladder, large epithelial cells with multiple cellular and nuclear features of atypia, Melamed‐Wolinska bodies, and invasion to some extent.^2^

In our case, the clinical history of UTI, undetected BRAF mutation, location of the lesions, patient age and gross and histological appearance of the lesions all were compatible with polypoid cystitis. However, we cannot rule out that CIS was already beginning to develop at the time when the first biopsy sample was taken. The apparent initial ultrasonographic improvement of the bladder after medical management in part may have been affected by operator variability and bladder volume.^28^, ^29^, ^30^

All types of UC consist of neoplastic transitional cell epithelium that is in various stages of differentiation.^2^ Carcinoma in situ is the most well‐differentiated form, and it is accepted by some pathologists that if CIS or low‐grade UC are the only lesions present in biopsy specimens, a more invasive neoplasm likely is present in different regions that may not have been included in the biopsy.^2^ Carcinoma in situ is a recognized precursor to invasive UC in humans, and therefore a similar progression may occur in animals.^2^

In our patient, the mutation in exon 15 of the canine BRAF gene present in >85% of TCC cases in dogs^20^ was not present. This characteristic complicated the final diagnosis for a substantial period of time and prevented confirmation of the time at which a malignant transformation occurred. This case emphasizes that, despite the high sensitivity of the CADET BRAF^19^ and CADET BRAF Plus,^20^ the results of these tests are limited to the analysis of a very small number of cells shed in the urine. Histopathology of the lesion remains the gold standard for identification of suspected TCC, without detection of the mutation.

After the confirmed diagnosis of TCC on cystoscopic biopsy, partial cystectomy was performed. Analysis of the full thickness bladder wall allowed us to confirm the diagnosis of TCC despite the previously conflicting results of cystoscopic biopsies and undetected BRAF mutation. Surgery also presumably alleviated the discomfort related to the chronically inflamed and neoplastic tissue. A recent study in dogs with bladder TCC found the best outcomes (median survival time, 772 days) for dogs with nontrigonal bladder TCC treated by full thickness partial cystectomy and daily piroxicam treatment, with or without chemotherapy.^31^ At the time of writing, our patient is still alive and has a normal appearing bladder and urethra on ultrasound examination, 431 and 180 days after first diagnosis of TCC and partial cystectomy, respectively.

This report should increase the awareness of potential rapid malignant transformation from polypoid cystitis into TCC. Histopathology is the gold standard for final diagnosis when the BRAF mutation is not detected, and TCC remains a differential diagnosis. Surgical management should be considered if rapid resolution is not achieved with medical management.

## CONFLICT OF INTEREST DECLARATION

Authors declare no conflict of interest.

## OFF‐LABEL ANTIMICROBIAL DECLARATION

Authors declare no off‐label use of antimicrobials.

## INSTITUTIONAL ANIMAL CARE AND USE COMMITTEE (IACUC) OR OTHER APPROVAL DECLARATION

Authors declare no IACUC or other approval was needed because this was retrospective.

## HUMAN ETHICS APPROVAL DECLARATION

Authors declare human ethics approval was not needed for this study.
